# The Effect of Gate Work Function and Electrode Gap on Wide Band-Gap Sn-Doped α-Ga_2_O_3_ Metal–Semiconductor Field-Effect Transistors

**DOI:** 10.3390/ma15030913

**Published:** 2022-01-25

**Authors:** Han-Sol Ro, Sung Ho Kang, Sungyeop Jung

**Affiliations:** 1 Semiconductor Devices and Circuits Laboratory, Advanced Institute of Convergence Technology, Seoul National University, Suwon 16229, Korea; hansolro@snu.ac.kr; 2 Research Center for Materials, Components and Equipment, Advanced Institute of Convergence Technology, Seoul National University, Suwon 16229, Korea; bendo@snu.ac.kr

**Keywords:** metal–semiconductor field-effect transistors, work function, device structure, technology computer-aided design, numerical simulation

## Abstract

We present technology computer aided design (TCAD) results for wide band-gap Sn-doped α-Ga_2_O_3_ metal–semiconductor field-effect transistors (MESFETs). In particular, the effect of gate work function and electrode gap length on the electrical characteristics is demonstrated for a thorough understanding of the behavior of such devices. The gate work function significantly affects the reverse bias drain current under the gate-current dominant regime, whereas a gate-source/drain gap larger than 0.1 µm has a negligible effect on the drain current.

## 1. Introduction

During the past decade, ultrawide bandgap Ga_2_O_3_ semiconductors with a bandgap of 4.5~5.3 eV (depending on the crystal structure) have been investigated as an alternative to SiC and GaN (3.3 and 3.4 eV, respectively) for high-power electronic device applications [1,2]. Among five different phases of Ga_2_O_3_ (α, β, γ, ε, and δ) [3], the orthorhombic β phase is the most stable thermodynamically, while the rhombohedral corundum α phase is semi-stable [4]. On the other hand, the band gap of α-Ga_2_O_3_ is 5.3 eV [5,6], which is wider compared to β-Ga_2_O_3_ 4.9 eV [1,7,8], promising a higher breakdown field.

Conventionally, β-Ga_2_O_3_ has been grown via molecular beam epitaxy [1,2] on a β-Ga_2_O_3_ substrate grown from the melt [9]. However, it is difficult to produce a β-Ga_2_O_3_ wafer with a diameter large enough for practical application due to easy formation of cleavages such that the wafer size is limited to four inches [9]. Recently, mist chemical vapor deposition (Mist-CVD) has been introduced as a non-vacuum solution-process heteroepitaxy for α-Ga_2_O_3_ on mass-produced sapphire (Al_2_O_3_) wafers up to six inches, with a similar a crystal structure to α-Ga_2_O_3_ [10,11,12,13,14]. Being able to lift α-Ga_2_O_3_ off of the sapphire substrate and bond it to other substrates with high thermal conductivity (such as SiC, AlN, diamond, etc.) provides an additional advantage in high power switching and RF applications over β-Ga_2_O_3_ with a low thermal conductivity [9]. Despite these promising results on the epitaxial growth of α-Ga_2_O_3_ on sapphire, there are few demonstrations of electronic devices based on α-Ga_2_O_3_ [15,16,17].

A high-quality Silver oxide AgO_x_ Schottky contact was incorporated into Sn-doped α-Ga_2_O_3_ metal-semiconductor field-effect transistors (MESFET) [16] in order to achieve high rectifying Schottky contact at the gate–semiconductor interface. The use of the non-metallic gate electrode for the α-Ga_2_O_3_ MESFET enables the formation of the gate electrode and the metallic source/drain contact at the same plane (i.e., a coplanar configuration). In 2019, an in-depth experimental study on the oxidized metal Schottky contacts (of which the work function ranges from 4.70 to 5.80 eV) including AgO_x_, on β-Ga_2_O_3_ was reported [18]. However, a similar work on the oxidized metal Schottky contacts on α-Ga_2_O_3_ has not been reported yet. Therefore, the design strategy for optimal operation is lacking.

In this study, we begin by conducting a study on the effect of the gate work function on the electrical characteristics of wide band-gap Sn-doped α-Ga_2_O_3_ MESFET for a broad range of the work function, from 4.40 to 5.80 eV. The optimal gate work function found thusly will be applied while varying the source/drain-gate gap length between 0.1 to 2.0 µm. Electrical characteristics issued from these parameters will then be discussed, deepening our knowledge of the optimal configuration of such a device.

## 2. Materials and Methods

### 2.1. Metal–Semiconductor Field-Effect Transistor (MESFET)

**Figure 1:**
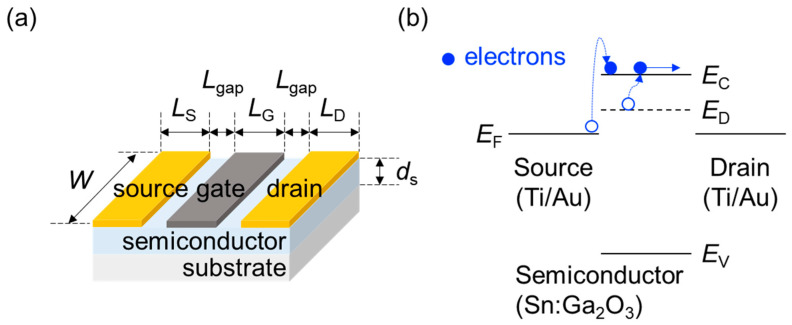
(**a**) A schematic diagram for the device structure of an Sn-doped α-Ga_2_O_3_ MESFET; (**b**) the energy diagram of the corresponding device.

A metal–semiconductor field-effect transistor (MESFET) consists of a substrate, a semiconductor layer, the gate electrode (G), and the source (S) and drain (D) electrodes (Figure 1). For a coplanar structure, the channel length *L* is defined as the distance between the S and D electrode; hence, *L* = *L*_G_ + 2 × *L*_gap_, where *L*_G_ is the gate length and *L*_gap_ is the gap between the S/D electrode and the G electrode. The channel width is denoted by *W*. The thickness of the semiconductor layer is denoted by *d*_s_.

The energy structure of an *n*-type MESFET is determined by the conduction and the valence band edge level, *E*_C_ and *E*_V_; the total density of states for the conduction and valence band of the semiconductor, *N*_C_ and *N*_V_; the donor level, *E*_D_; the total density of states for donor *N*_D_ of the n-type dopant; and the work function of S/D and G, *W*_S/D_ and *W*_G_, respectively. The dielectric constant *ε*_s_, electron and hole mobility, respectively *μ*_e_ and *μ*_h_, and electron effective mass, *m*_e_, describe the electrical properties of the semiconductor.

A complete list of parameters used for the simulation is provided in Table 1. The values correspond to the Sn-doped α-Ga_2_O_3_ MESFET with Ti as the S/D electrodes and AgO_x_ as the gate electrode. α-Ga_2_O_3_ is amenable to n-type doping by Sn as well [10,19,20], which enhances the free electron concentration and hence the mobility, and facilitates charge carrier injection at the source/drain. The values for *E*_D_ and *N*_D_ were taken from [16]. The typical value and the range of *W*_G_ were determined considering the reported values in [18,21]. The value for *W*_S/D_ was taken from [22]. Note that edge dislocation could present in the α-Ga_2_O_3_ epitaxy layer, around 10^7^ (epitaxial lateral overgrowth) ~ 10^10^ cm^−2^ (Mist-CVD), depending on the deposition methods [9], which lowers electron mobility, i.e., 1.3 cm^2^V^−1^s^−1^ for high edge dislocation density [5,15,23] and 24 cm^2^V^−1^s^−1^ for low edge dislocation density [24] compared to the theoretical value 300 cm^2^V^−1^s^−1^. In this study, the effect of dislocation is considered by carrier mobility.

Although there is a lack of study on the effect of defects at the interface between α-Ga_2_O_3_ and metal/oxidized metal, for oxide semiconductors the most likely defects are oxygen vacancies, *V*_O_, formed by chemical reactions during metal deposition [25,26]. The number of *V*_O_ is smaller at the semiconductor–oxidized metal interface compared to the semiconductor–metal interface because of the oxygen-rich deposition conditions for oxidized metal layer [18], which is likely to prevent Fermi level pinning by *V*_O_. Therefore, in this study, the effect of Fermi level pinning is not considered at the semiconductor–gate interface.

### 2.2. Numerical Simulation

The numerical simulation of MESFET resolves the coupled drift–diffusion current equation and the Poisson’s equation to obtain the current–voltage characteristics and the current density, charge carrier, and potential distribution. We adopted TCAD software Atlas from Silvaco, Santa Clara, CA, USA. [27]. It is an advantage of numerical simulation that the work function can be varied without altering other physical parameters, which is difficult to achieve experimentally. We considered the Schottky barrier lowering and tunneling models computed by Wentzel-Kramers-Brillouin approximation [28] at both the source/drain-semiconductor and the gate–semiconductor junction. This allows description of the charge carrier injection at the Schottky junction with a large injection barrier.

In order to investigate the effect of the gate work function, *W*_G_, we varied the latter from 4.4 to 5.8 eV by 0.2 eV while fixing the source/drain-gate gap to 1.0 µm and the gate length to 8.0 µm. Then, in order to investigate the effect of the source/drain-gate gap, *L*_gap_, *L*_gap_ was varied as 0.1, 0.2, 0.5, 1.0, and 2.0 µm. Concomitantly, the gate length changed accordingly, as 9.8, 9.6, 9.0, 8.0, and 6.0 µm, as the channel length *L* was fixed to 10 µm. The gate work function *W*_G_ for the second simulation set was fixed at 5.4 eV.

## 3. Results and Discussion

### 3.1. Effect of Gate Work Function Variation on Sn-Doped α-Ga_2_O_3_ Metal–Semiconductor Field-Effect Transistors

#### 3.1.1. Current–Voltage (I-V) Characteristics

**Figure 2:**
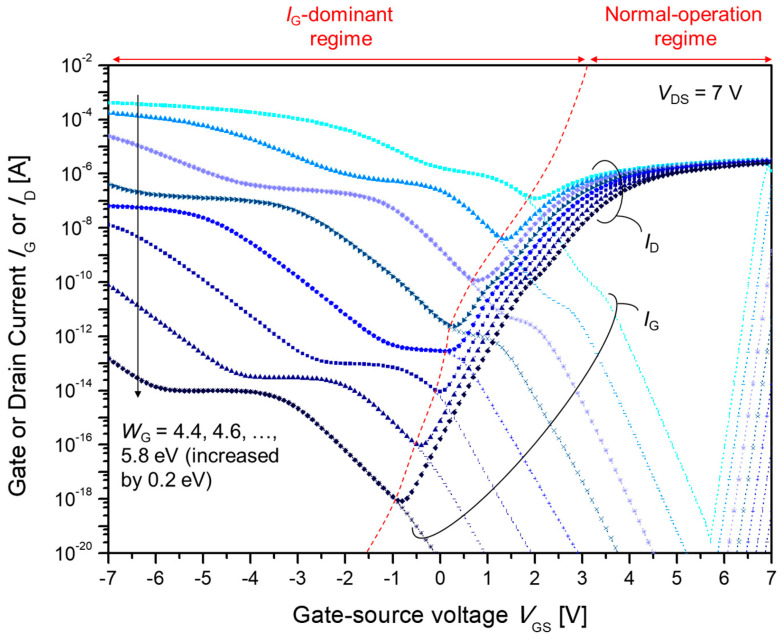
Simulated gate current, *I*_G_, and drain current, *I*_D_ for various gate work functions *W*_G_. *W*_G_ was varied from 4.4 to 5.8 eV by 0.2 eV. An *I*_G_-dominant region is observed for the gate voltages smaller than the on voltage *V*_GS_ < *V*_on_.

Figure 2 shows the simulated current–voltage (*I*-*V*) characteristics of MESFETs with various gate work functions. The gate current *I*_G_ and drain current I_D_ are plotted as a function of the gate-source voltage *V*_GS_. When *V*_GS_ is larger than the on voltage, *V*_on_, and smaller than a certain voltage (~7 V, which is similar to the drain-source, *V*_DS_), *V*_on_ < *V*_GS_ ⪅ *V*_DS_ and *I*_D_ dominates over *I*_G_. When *V*_GS_ ⪆ *V*_DS_, *I*_G_ dominates over *I*_D_ because the drain-gate diode is now forward biased. When *V*_GS_ is smaller than the on voltage *V*_on_, *I*_G_ is a dominant factor.

The on-off ratio, defined as the ratio of *I*_D_ at *V*_GS_ = 7 V to that at *V*_GS_ = −7 V, increases as the gate Fermi level is lowered. When *W*_G_ ≤ 5.0 eV, the drain current *I*_D_ under the *I*_G_-dominant regime becomes comparable and even larger than that under the normal-operation regime. Therefore, the device could not be used as a switching element. When *W*_G_ > 5.0 eV, the on-off ratio is around 10^1^ ~ 10^7^, showing good rectification behavior. In summary, the degree of electron injection into the gate electrode on the drain side, as will be shown in the following sections, determines the level of off-current, and hence the on-off ratio of the transistor.

In addition, *V*_on_ should be as close as possible to 0 V to guarantee functional transistor behavior. Thus, a gate work function of *W*_G_ = 5.4 eV is the optimal condition. This condition was used to analyze the effect of the source/drain-gate gap *L*_gap_.

#### 3.1.2. Current Density Distribution and Vector

**Figure 3:**
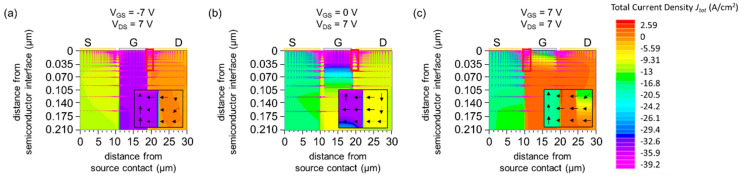
Simulated total current density *J*_tot_(x, y) for (**a**) *V*_GS_ = −7 V and *V*_DS_ = 7 V (*I*_G_-dominant regime), (**b**) *V*_GS_ = 0 V and *V*_DS_ = 7 V (off regime), (**c**) *V*_GS_ = 7 V and *V*_DS_ = 7 V (normal-operation regime). The arrows represent the simulated total current density vector. A magnified view of the semiconductor region near the gate electrode (18 µm ≤ x ≤ 20 µm for (**a**,**b**) and 9 µm ≤ x ≤ 11 µm for (**c**)) is shown for (**a**–**c**). The work function of the gate is *W*_G_ = 5.4 eV. The red boxes indicate the region where the total current density is high.

Figure 3a,b provides direct evidence that the current flows into the gate electrode under the *I*_G_-dominant regime and the off regime. In particular, the current density is high at the edge of gate on the drain side (black boxes). On the other hand, the current flows out from the gate electrode (Figure 3c). The current coming from the drain joins that coming from the gate, and flows into the source, which establishes the current path of the device under the normal-operation regime.

#### 3.1.3. Carrier Concentration and Potential Distribution

**Figure 4:**
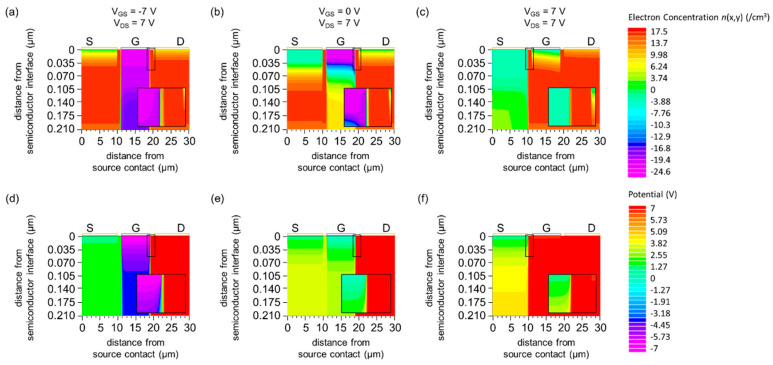
(**a**–**c**) Simulated carrier concentration distribution for *n*(*x*, *y*) (**a**) *V*_GS_ = −7 V and *V*_DS_ = 7 V (*I*_G_-dominant regime), (**b**) *V*_GS_ = 0 V and *V*_DS_ = 7 V (off regime), (**c**) *V*_GS_ = 7 V and *V*_DS_ = 7 V (normal-operation regime). (**d**–**f**) Simulated potential distribution *V*(*x*, *y*) for (**d**) *V*_GS_ = −7 V and *V*_DS_ = 7 V, (**e**) *V*_GS_ = 0 V and *V*_DS_ = 7 V, (**f**) *V*_GS_ = 7 V and *V*_DS_ = 7 V. The entire semiconductor layer is shown for all panels (**a**–**f**). A magnified view of the semiconductor region near the gate electrode (18 µm ≤ x ≤ 20 µm for (**a**,**b**,**d**,**e**) and 9 µm ≤ x ≤ 11 µm for (**c**,**f**)) is shown for all panels. The work function of the gate is *W*_G_ = 5.4 eV.

The semiconductor under the gate electrode is fully or partially depleted, whereas the semiconductor under the source/drain-gate gap is accumulated and the charge carrier concentration is high (*n* ~ 10^17^ cm^−3^ ) (Figure 4a–c). In addition, the potential difference is −7 V between G and S and −14 V between G and D (under the I_G_-dominant regime, Figure 4d), and 0 V between G and S and −7 V between G and D (under the off regime, Figure 4e). Thus, the current flows into the gate under the *I*_G_-dominant regime and the off regime.

### 3.2. Effect of Source/Drain-Gate Gap Variation on Sn-Doped α-Ga_2_O_3_ Metal–Semiconductor Field-Effect Transistors

#### 3.2.1. Current–Voltage (I-V) Characteristics

**Figure 5:**
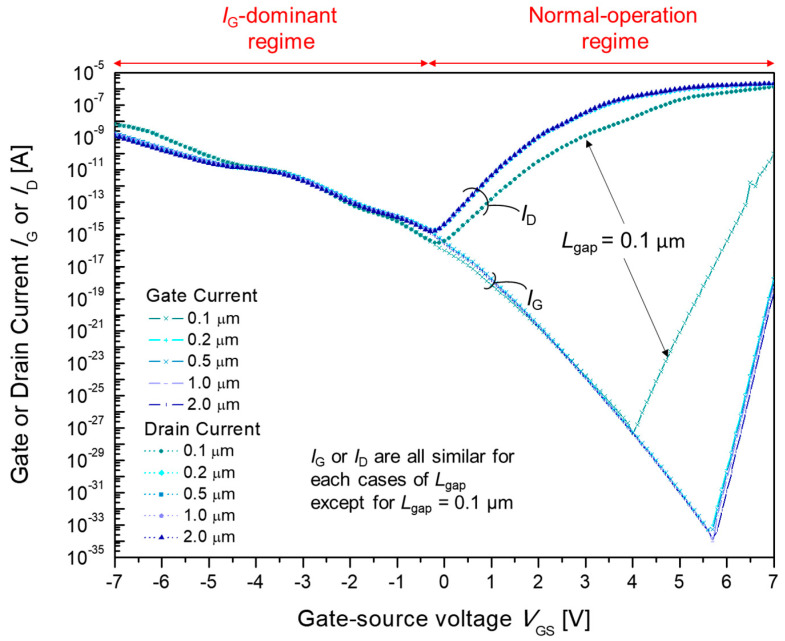
Simulated gate current *I*_G_ and drain current *I*_D_ for various source/drain-gate gaps, *L*_gap_. *L*_gap_ was divided into five separate cases of 0.1, 0.2, 0.5, 1.0 and 2.0 µm.

In general, the current–voltage characteristics for all cases of *L*_gap_ between 0.1 to 2.0 µm (shown in Figure 5) feature the typical *I*-*V* characteristics of MESFET, with a greater *I*_D_ compared to *I*_G_ when *V*_GS_ is higher than *V*_on_ and a greater *I*_G_ compared to *I*_D_ when *V*_GS_ is lower than *V*_on_.

In detail, *I*_D_ decreases as *L*_gap_ increases in the *I*_G_-dominant regime, whereas *I*_D_ increases as *L*_gap_ increases in the normal-operation regime. However, *I*_G_ decreases as *L*_gap_ increases in both the *I*_G_-dominant regime and the normal-operation regime. It is noticeable that the *I*-*V* characteristics for *L*_gap_ = 0.1 µm are significantly different, with a longer *L*_gap_ = 0.2, 0.5, 1.0, and 2.0 µm. Such differences are explained in the following sections by considering the current path of the device with the current density, charge concentration and potential distribution. It can be inferred that under the normal operation regime electron transport under the gate–source gap does not deteriorate the current unless the carrier concentration under the gap is maintained at a high enough level.

#### 3.2.2. Current Density Distribution and Vector

**Figure 6:**
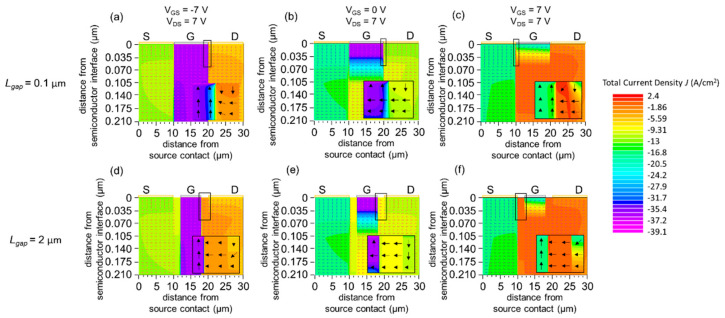
(**a**–**c**) Simulated total current density *J*_tot_(x, y) for *L*_gap_ = 0.1 µm (**a**) *V*_GS_ = −7 V and *V*_DS_ = 7 V (*I*_G_-dominant regime), (**b**) *V*_GS_ = 0 V and *V*_DS_ = 7 V (off regime), (**c**) *V*_GS_ = 7 V and *V*_DS_ = 7 V (normal-operation regime). (**d**–**f**) Simulated total current density *J*_tot_(x, y) for *L*_gap_ = 2.0 µm (**d**) *V*_GS_ = −7 V and *V*_DS_ = 7 V (*I*_G_-dominant regime), (**e**) *V*_GS_ = 0 V and *V*_DS_ = 7 V (off regime), (**f**) *V*_GS_ = 7 V and *V*_DS_ = 7 V (normal-operation regime). The arrows represent the simulated total current density vector. A magnified view of the semiconductor region near the gate electrode (19.8 µm ≤ x ≤ 20.1 µm for (**a**,**b**), 9.9 µm ≤ x ≤ 10.2 µm for (**c**), 17 µm ≤ x ≤ 21 µm for (**d**,**e**), 9 µm ≤ x ≤ 13 µm for (**f**)) is shown for all panels. The work function of the gate is *W*_G_ = 5.4 eV. The black boxes indicate the region where the total current density is high.

Figure 6 shows the current density distribution and its vector in the *I*_G_-dominant regime, off regime, and normal-operation regime for *L*_gap_ = 0.1 µm (Figure 6a–c) and *L*_gap_ = 2.0 µm (Figure 6d–f). As discussed in Section 3.2.1, the current flows into the gate electrode from the drain electrode under the *I*_G_-dominant regime and the off regime. For both values of *L*_gap_, the current density is high at the edge of the gate on the drain side (highlighted by the black rectangle) under the *I*_G_-dominant regime and the off regime. In the normal-operation regime, the drain current joins the gate current to flow into the source.

The differences in current–voltage characteristics between *L*_gap_ = 0.1 µm and the other cases can be elucidated by the current path. In the *I*_G_-dominant regime, the current flows from the source and drain electrodes toward the gate electrodes. Therefore, a smaller gap length between the gate and source/drain electrodes increases both *I*_D_ and *I*_G_ by providing a shorter resistive path to the gate electrode. In the case of the off regime, the current flows from the drain electrode to the source electrode while being leaked in the gate channel area. A smaller *L*_gap_ decreases both *I*_D_ and *I*_G_ due to a longer gate current path between the drain and source electrodes. Noticeably, a greater difference in the drain current *I*_D_ compared to *I*_G_ undermines leakage of the drain current while crossing the gate channel area. Lastly, in the normal-operation regime the current flows from the drain and gate electrodes toward the source electrode. Therefore, there is no crowding of current at the frontier of the gate electrode and drain–gate electrode gap. Due to this phenomenon, the current density *I*_D_ remains almost constant when *L*_gap_ > 0.1 µm. Meanwhile, the gate current *I*_D_ shows a drastic difference in cases where *L*_gap_ is 0.1 µm. This could originate from the fact that the current from both the gate and the drain accumulates itself at the edge of the source electrode. A more plausible explanation can be made by referring to the charge carrier concentration and potential distribution, as detailed in the following section.

#### 3.2.3. Carrier Concentration and Potential Distribution

**Figure 7:**
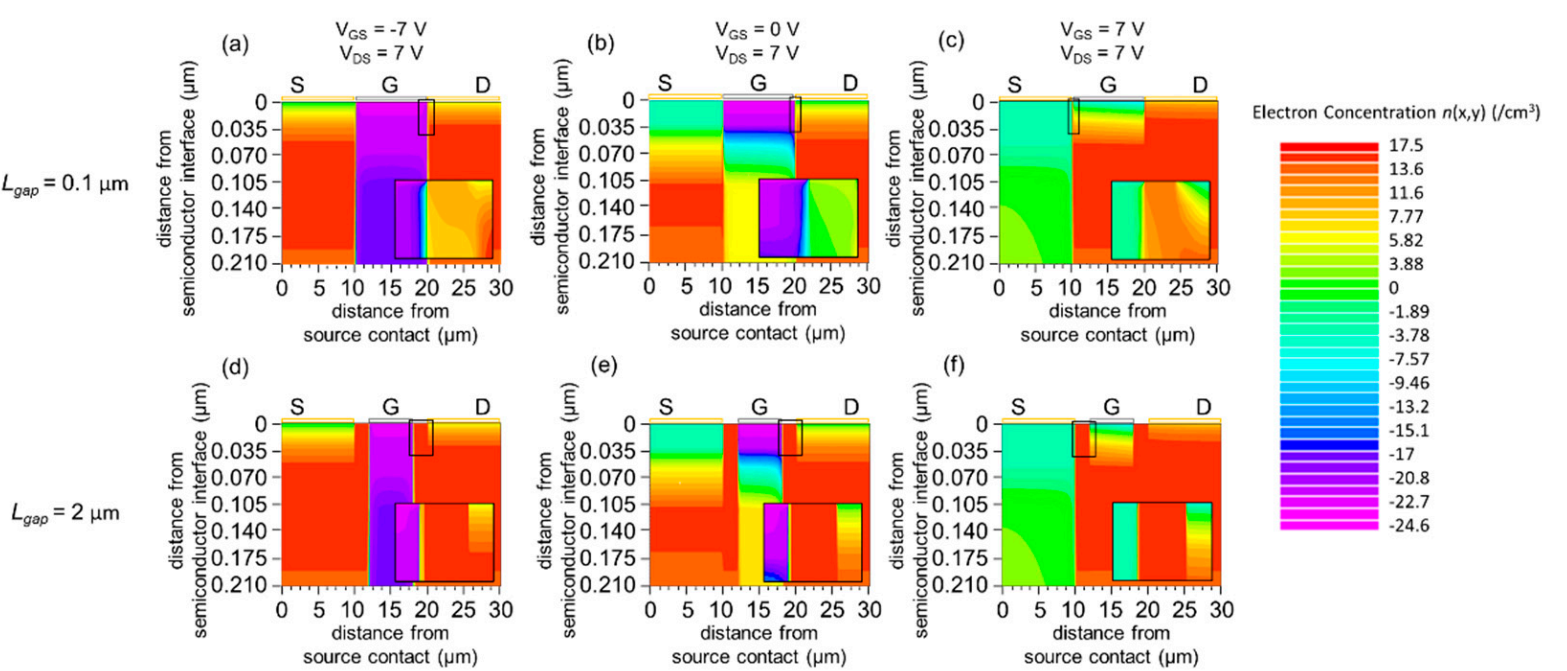
(**a**–**c**) Simulated carrier concentration distribution *n*(*x*, *y*) for *L*_gap_ = 0.1 µm (**a**) *V*_GS_ = −7 V and *V*_DS_ = 7 V (*I*_G_-dominant regime), (**b**) *V*_GS_ = 0 V and *V*_DS_ = 7 V (off regime), (**c**) *V*_GS_ = 7 V and *V*_DS_ = 7 V (normal-operation regime). (**d**–**f**) Simulated carrier concentration distribution *n*(*x*, *y*) for *L*_gap_ = 2.0 µm (**d**) *V*_GS_ = −7 V and *V*_DS_ = 7 V (*I*_G_-dominant regime), (**e**) *V*_GS_ = 0 V and *V*_DS_ = 7 V (off regime), (**f**) *V*_GS_ = 7 V and *V*_DS_ = 7 V (normal-operation regime). The entire semiconductor layer is shown for all panels (**a**–**f**). A magnified view of the semiconductor region near the gate electrode (19.8 µm ≤ x ≤ 20.1 µm for (**a**,**b**), 9.9 µm ≤ x ≤ 10.2 µm for (**c**), 17 µm ≤ x ≤ 21 µm for (**d**,**e**), 9 µm ≤ x ≤ 13 µm for (**f**)) is shown for all panels. The work function of the gate is *W*_G_ = 5.4 eV.

The simulation results of the carrier concentration distribution *n*(*x*, *y*), shown in Figure 7, reveal that the semiconductor under the gate electrode is either fully depleted in the *I*_G_-dominant regime or partially depleted in the off regime and normal-operation regime for both cases of *L*_gap_. On the other hand, a high carrier concentration up to ~10^17^ cm^−3^ is observed beneath the electrodes gap, where the effect of the gate field is out of reach. This phenomenon is more pronounced in the case of a larger *L*_gap_. For *L*_gap_ = 0.1 µm when the gap becomes comparable to a few Debye length, the effect of the gate field is present in the gap, as reported in [29]. In this case, the carrier concentration in the gap becomes approximately 10^6^ cm^−3^ lower than 10^17^ cm^−3^ by several orders of magnitude.

**Figure 8:**
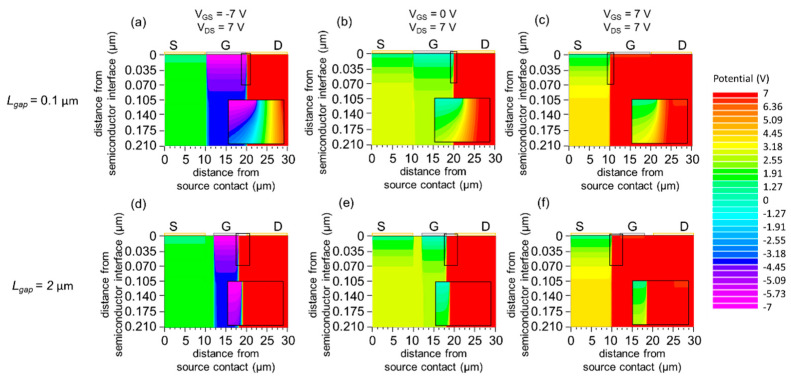
(**a**–**c**) Simulated potential distribution *V*(*x*, *y*) for *L*_gap_ = 0.1 µm (**a**) *V*_GS_ = −7 V and *V*_DS_ = 7 V (*I*_G_-dominant regime), (**b**) *V*_GS_ = 0 V and *V*_DS_ = 7 V (off regime), (**c**) *V*_GS_ = 7 V and *V*_DS_ = 7 V (normal-operation regime). (**d**–**f**) Simulated potential distribution *V*(*x*, *y*) for *L*_gap_ = 2.0 µm (**d**) *V*_GS_ = −7 V and *V*_DS_ = 7 V (*I*_G_-dominant regime), (**e**) *V*_GS_ = 0 V and *V*_DS_ = 7 V (off regime), (**f**) *V*_GS_ = 7 V and *V*_DS_ = 7 V (normal-operation regime). The entire semiconductor layer is shown for all panels (**a**–**f**). A magnified view of the semiconductor region near the gate electrode (19.8 µm ≤ x ≤ 20.1 µm for (**a**,**b**), 9.9 µm ≤ x ≤ 10.2 µm for (**c**), 17 µm ≤ x ≤ 21 µm for (**d**,**e**), 9 µm ≤ x ≤ 13 µm for (**f**)) is shown for all panels. The work function of the gate is *W*_G_ = 5.4 eV.

Figure 8 shows the potential distribution in the device for an electrode gap of *L*_gap_ = 0.1 and 2.0 µm. Similar to the observation in Section 3.1.3, the greater potential difference between gate and drain under the *I*_G_-dominant regime (Figure 8a,d) and off regime (Figure 8b,e) justifies the high current density concentration at the edge of the gate electrode from the drain electrode. In the normal-operation regime (Figure 8c,f), a greater potential difference is found at the edge of the source electrode from the gate electrode. Thereby, the high current density flows in this area.

## 4. Conclusions

In this study, we have described the effects of the gate work function and electrode gap on the electrical characteristics of Sn-doped α-Ga_2_O_3_ MESFETs using TCAD software. The gate work function significantly changes the current level of the *I*_G_-dominant regime, hence the rectification ratio. The existence and the mechanism of the gate current under the *I*_G_-dominant regime were illustrated by simulated current density distribution and vector as well as by charge carrier and potential distribution, allowing for determination of a theoretical optimal gate work function value of a coplanar MESFET. As for the electrode gap, the simulation results of the current vector enabled us to understand the current path in Sn-doped α-Ga_2_O_3_ MESFETs. It is imperative to respect a certain amount of gap distance between electrodes of at least than 0.1 µm to prevent the effect of the gate field in the gap region. Considering that most research efforts have been focused on the deposition and characterization of an Sn-doped α-Ga_2_O_3_ heteroepitaxial layer, this study on device simulation will help to translate such knowledge concerning α-Ga_2_O_3_ heteroepitaxy into device design, fabrication and optimization for further improvement of device performance.

## Figures and Tables

**Table 1:** Parameters used for TCAD Simulation.

Name	Symbol	Value	Unit
Channel length	*L*	10	µm
Gate length	*L*_G_	6.0~9.8	µm
Source/drain-gate gap	*L*_gap_	0.1~2.0	µm
Source and drain length	*L*_S/D_	10	µm
Channel width	*W*	262	µm
Semiconductor thickness	*d*_s_	210	nm
Conduction band edge level	*E*_C_	3.00	eV
Valence band edge level	*E*_V_	7.30	eV
Total density of states for conduction band	*N*_C_	4.97 × 10^18^	cm^−3^
Total density of states for valence band	*N*_V_	4.97 × 10^18^	cm^−3^
Total density of states for donor	*N*_D_	3.00 × 10^17^	cm^−3^
Donor level	*E*_D_	1.10	eV
Source/drain work function	*W*_S/D_	4.33	eV
Gate work function	*W*_G_	4.40~5.80	eV
Semiconductor relative dielectric constant	*ε*_s_	10	-
Electron mobility	*μ*_e_	1.3	cm^2^V^−1^s^−1^
Hole mobility	*μ*_h_	1.3	cm^2^V^−1^s^−1^
Electron effective mass	*m*_e_	0.34	-

## Data Availability

The data presented in this study are available on request from the corresponding author.
